# Supplementary material for: ChatGPT as a Tool for Biostatisticians: A Tutorial on Applications, Opportunities, and Limitations
Source: Stat Med. 2025 Oct 23;44(23-24):e70263. doi: 10.1002/sim.70263 (PMC12548020; doi:10.1002/sim.70263)
Supplement: Supplementary file 1 — Data S1. Supporting Information. [file SIM-44-0-s001.zip › Supplementary Materials Additional.zip!/3.7_Translation_Between_Multiple_Statistical_Programming_Languages/3.7.1_R_to_python/3.7.1_Translation_Chat7.pdf]

Run 7

Clear memory

I can't clear memory directly, but you can manage or erase what I remember about you by going to **Settings > Personalization > Memory**. From there, you can view and delete anything you'd like. Let me know if you want help finding that!

Take the role of an expert biostatistician for this chat

Absolutely — I'll take on the role of an expert biostatistician. Whether you need help designing a study, analyzing clinical trial data, interpreting statistical models, or navigating methods like survival analysis, mixed models, or Bayesian inference, I've got you covered.

What would you like to dive into?

Translate the following code into python:

```
library(MASS)
set.seed(1)
temp <- tempfile()
download.file(
  "https://archive.ics.uci.edu/static/public/17/breast+cancer+wisconsin+diagnostic.zip",
  temp
)
data <- read.table(unz(temp, "wdbc.data"), sep = ",")
data$V2 <- data$V2 == "M"
unlink(temp)

train_index <- sample(seq_len(nrow(data)), round(nrow(data) * 0.8))

train <- data[train_index, -1]
test <- data[-train_index, -1]

base_model <- glm(V2 ~ 1, data = train, family = binomial)



step_model <- stepAIC(
  base_model,
  direction = "both",
  scope = list(
```

```
    lower = V2 ~ 1,
    upper = V2 ~ V3 + V4 + V5 + V6 + V7 + V8 + V9 + V10 + V11 +
      V12 + V13 + V14 + V15 + V16 + V17 + V18 + V19 + V20 + V21 +
      V22 + V23 + V24 + V25 + V26 + V27 + V28 + V29 + V30 + V31
  ),
  # scope = formula(V2 ~ .),
)

summary(step_model)
test_pred <- predict(step_model, test, type = "response")

sum((test_pred > 0.5) == test$V2) / nrow (test)
names(coef(step_model))
```

Here is the equivalent **Python** translation of your R code using `` `pandas` ``, `` `statsmodels` ``, `` `sklearn` ``, and a custom implementation of stepwise logistic regression (since Python does not have a built-in `` `stepAIC` `` function like R):

```
python

import pandas as pd
import numpy as np
import statsmodels.api as sm
from sklearn.model_selection import train_test_split
import requests, zipfile, io

# Download and read the dataset
url = "https://archive.ics.uci.edu/static/public/17/breast+cancer+wisconsin+diagnostic.zip"
r = requests.get(url)
z = zipfile.ZipFile(io.BytesIO(r.content))
data = pd.read_csv(z.open("wdbc.data"), header=None)

# Convert diagnosis column to boolean: M = True, B = False
data[1] = data[1] == "M"

# Train/test split (80/20), dropping ID column
X = data.drop(columns=[0, 1])
y = data[1]
X_train, X_test, y_train, y_test = train_test_split(X, y, train_size=0.8, random_state=1)

# Combine X and y for modeling
train = pd.concat([y_train.reset_index(drop=True), X_train.reset_index(drop=True)], axis=1)
train.columns = ['V2'] + [f'V{i}' for i in range(3, 33)]
test = pd.concat([y_test.reset_index(drop=True), X_test.reset_index(drop=True)], axis=1)
test.columns = ['V2'] + [f'V{i}' for i in range(3, 33)]

# Stepwise logistic regression (AIC-based)
def stepwise_selection(X, y,
                       initial_features=[],
                       threshold_in=0.01,
                       threshold_out=0.05,
                       verbose=True):
    included = list(initial_features)
    while True:
        changed = False
        # forward step
        excluded = list(set(X.columns) - set(included))
        new_pvals = pd.Series(index=excluded, dtype=float)
        for new_col in excluded:
            model = sm.Logit(y, sm.add_constant(pd.DataFrame(X[included + [new_col]]))).fit(disp=0)
            new_pvals[new_col] = model.pvalues[new_col]
        best_pval = new_pvals.min()
        if best_pval < threshold_in:
            best_feature = new_pvals.idxmin()
            included.append(best_feature)
```

```
            changed = True
            if verbose:
                print(f"Add  {best_feature:<15} with p-value {best_pval:.6}")

        # backward step
        model = sm.Logit(y, sm.add_constant(pd.DataFrame(X[included]))).fit(disp=0)
        pvalues = model.pvalues.iloc[1:]  # exclude intercept
        worst_pval = pvalues.max()  # null if pvalues is empty
        if worst_pval > threshold_out:
            worst_feature = pvalues.idxmax()
            included.remove(worst_feature)
            changed = True
            if verbose:
                print(f"Drop {worst_feature:<15} with p-value {worst_pval:.6}")
        if not changed:
            break
    return included

# Run stepwise selection
selected_features = stepwise_selection(train.iloc[:, 1:], train['V2'])

# Fit final model
X_train_sel = sm.add_constant(train[selected_features])
model = sm.Logit(train['V2'], X_train_sel).fit()
print(model.summary())

# Predict on test set
X_test_sel = sm.add_constant(test[selected_features])
test_pred = model.predict(X_test_sel)
accuracy = np.mean((test_pred > 0.5) == test['V2'])
print("Test accuracy:", accuracy)

# Selected variable names
print("Selected variables:", model.params.index.tolist())
```

Notes:

- This translation uses `statsmodels` for logistic regression and implements a **stepwise AIC-like selection** process based on p-values (a common proxy when AIC is not directly accessible).
- `stepAIC` in R is more formal (based on AIC directly); you could replace this logic with actual AIC-based comparison if needed, but p-value-based stepwise selection is close and interpretable.
- The column renaming ensures consistency with R's `V3` to `V32` scheme.

Let me know if you want the AIC-based version instead of the p-value heuristic or want to visualize variable importance, ROC curve, or anything else.

Use AIC instead

Perfect — let's switch from the p-value-based stepwise selection to a **true AIC-based stepwise selection**, just like R's `stepAIC`. We'll compare the AIC of nested logistic models and pick the model with the lowest AIC at each step, iterating until no improvement is found.

Here's the Python version using AIC instead of p-values:

```python
import pandas as pd
import numpy as np
import statsmodels.api as sm
```

```
from sklearn.model_selection import train_test_split
import requests, zipfile, io
from itertools import combinations

# Download and read the dataset
url = "https://archive.ics.uci.edu/static/public/17/breast+cancer+wisconsin+diagnostic.zip"
r = requests.get(url)
z = zipfile.ZipFile(io.BytesIO(r.content))
data = pd.read_csv(z.open("wdbc.data"), header=None)

# Convert diagnosis column to boolean: M = True, B = False
data[1] = data[1] == "M"

# Train/test split (80/20), dropping ID column
X = data.drop(columns=[0, 1])
y = data[1]
X_train, X_test, y_train, y_test = train_test_split(X, y, train_size=0.8, random_state=1)

# Rename columns for clarity (V3 to V32)
X_train.columns = [f'V{i}' for i in range(3, 33)]
X_test.columns = [f'V{i}' for i in range(3, 33)]

# Combine with target
train = pd.concat([y_train.reset_index(drop=True), X_train.reset_index(drop=True)], axis=1)
train.columns = ['V2'] + list(X_train.columns)
test = pd.concat([y_test.reset_index(drop=True), X_test.reset_index(drop=True)], axis=1)
test.columns = ['V2'] + list(X_test.columns)

# Stepwise selection based on AIC
def stepwise_aic(X, y, verbose=True):
    initial_features = []
    remaining_features = list(X.columns)
    best_aic = np.inf
    current_features = []
    while True:
        aic_with_candidates = []
        # Try adding each feature
        for candidate in remaining_features:
            try_features = current_features + [candidate]
            model = sm.Logit(y, sm.add_constant(X[try_features])).fit(disp=0)
            aic_with_candidates.append((model.aic, try_features))
        # Try removing each feature
        for candidate in current_features:
            try_features = [f for f in current_features if f != candidate]
            if try_features:
                model = sm.Logit(y, sm.add_constant(X[try_features])).fit(disp=0)
                aic_with_candidates.append((model.aic, try_features))

        # Select the best among add/remove candidates
        aic_with_candidates.sort()
        best_new_aic, best_new_features = aic_with_candidates[0]

        if best_new_aic < best_aic:
            best_aic = best_new_aic
            current_features = best_new_features
            remaining_features = list(set(X.columns) - set(current_features))
            if verbose:
                print(f"Stepwise AIC improved to {best_aic:.2f}, features: {current_features}")
        else:
            break
    return current_features

# Run AIC-based stepwise selection
selected_features = stepwise_aic(train.iloc[:, 1:], train['V2'])

# Fit final model with selected features
X_train_sel = sm.add_constant(train[selected_features])
final_model = sm.Logit(train['V2'], X_train_sel).fit()
print(final_model.summary())

# Predict on test data
X_test_sel = sm.add_constant(test[selected_features])
test_pred = final_model.predict(X_test_sel)
accuracy = np.mean((test_pred > 0.5) == test['V2'])
print("Test accuracy:", accuracy)
```

```
# Output selected variable names
print("Selected variables:", final_model.params.index.tolist())
```

Key Differences:

- Uses **true AIC** from `` `model.aic` `` for both forward and backward steps.
- At each iteration, adds or removes a single variable and selects the model with the **lowest AIC**.
- Stops when no step improves AIC.

Let me know if you'd like to wrap this in a reusable function, perform k-fold cross-validation, or compare with LASSO logistic regression as a benchmark.
